# Determination of the pathogenicity of known *COL4A5* intronic variants by *in vitro* splicing assay

**DOI:** 10.1038/s41598-019-48990-9

**Published:** 2019-09-03

**Authors:** Tomoko Horinouchi, Kandai Nozu, Tomohiko Yamamura, Shogo Minamikawa, China Nagano, Nana Sakakibara, Koichi Nakanishi, Yuko Shima, Naoya Morisada, Shinya Ishiko, Yuya Aoto, Hiroaki Nagase, Hiroki Takeda, Rini Rossanti, Hiroshi Kaito, Masafumi Matsuo, Kazumoto Iijima

**Affiliations:** 1 Department of Pediatrics, Kobe University Graduate School of Medicine, 7-5-1 Kusunoki-cho, Chuo, Kobe, Hyogo, 650-0017 Japan; 2 Department of Child Health and Welfare (Pediatrics), Graduate School of Medicine, University of the Ryukyus, 207, Uehara, Nishihara-cho, Tyutou, Okinawa, 903-0125 Japan; 3 Department of Pediatrics, Wakayama Medical University, 811-1, Kimiidera, Wakayama, Wakayama Prefecture 641-8510 Japan; 4 Department of Physical Therapy, Faculty of Rehabilitation, Kobe Gakuin University, 518, Arise, Ikawadani-cho, Nishi, Kobe, Hyogo, 651-2180 Japan

**Keywords:** Interstitial disease, Alport syndrome

## Abstract

X-linked Alport syndrome (XLAS) is a congenital renal disease caused by mutations in *COL4A5*. In XLAS cases suspected of being caused by aberrant splicing, transcript analysis needs to be conducted to determine splicing patterns and assess the pathogenicity. However, such analysis is not always available. We conducted a functional splicing assay using a hybrid minigene for seven *COL4A5* intronic mutations: one was identified by us and six were found in the Human Gene Mutation Database. The minigene assay revealed exon skipping in four variants, exon skipping and a 10-bp insertion in one variant, and no change in one variant, which appeared not to be pathogenic. For one variant, our assay did not work. The results of all three cases for which transcript data were available were consistent with our assay results. Our findings may help to increase the accuracy of genetic test results and clarify the mechanisms causing aberrant splicing.

## Introduction

*COL4A5* (NM: 000495.4) is the causative gene of X-linked Alport syndrome (XLAS). XLAS can cause end-stage renal disease accompanied by sensorineural hearing loss and ocular abnormalities in affected patients^1^. It has been reported that 13.7% or 14.9% of pathogenic *COL4A5* mutations are splice site mutations^2,3^. However, in these previous studies, no intronic variants leading to aberrant *COL4A5* splicing outside of the consensus sequence (AG-GT) were identified. Many unclassified pathogenic intronic or exonic mutations result in splicing abnormalities^4–6^. We previously reported that, of 41 families with *COL4A5* aberrant splicing, only 32 (78%) had splice site variants, while the others had deep intronic or exonic variants^5^. Clinically diagnosed XLAS cases lacking *COL4A5* exonic or consensus splice site variants can be caused by intronic or synonymous exonic splicing abnormalities.

Recently, we conducted a genetic analysis of inherited kidney diseases. However, it is sometimes difficult to distinguish intronic variants leading to splicing errors from harmless polymorphisms. The most reliable method to identify splicing aberrations is by *in vivo* assay. This method is based on the analysis of mRNA derived from the affected tissue. However, this sample type is not always available. Instead, we analyse mRNA isolated from peripheral blood leukocytes, which is unstable and easily destroyed during transportation. To prevent RNA destruction, we use the Paxgene Blood RNA Kit (Qiagen Inc., Chatsworth, CA) or RNAlater RNA Stabilization Reagent (Qiagen Inc.). However, even using these tools, it is sometimes difficult to extract mRNA of sufficient quantity and quality.

Several *in silico* approaches have been developed to assess the influence of sequence variants on splicing^7^, but the information acquired using these approaches remains incomplete^8,9^. They can provide information about changes in the ability of splicing-related proteins to bind to each splicing related element, but do not accurately predict splicing patterns. Recently, the *in vitro* splicing assay with hybrid minigene has emerged as a relatively fast approach to identify splicing aberrations in inherited kidney disease^10–13^. Three reports have shown that the hybrid minigene system, using HEK293 or HEK293T cells derived from human embolic kidney cells, was effective for assessing *COL4A5* variant splicing patterns^5,14,15^. Currently, the best approach to identify splicing mutations with a role in disease is to use a combination of these *in vivo*, *in silico* and *in vitro* analyses^7^.

**Figure 1:**
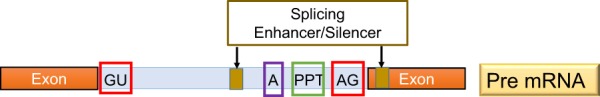
Schema for splicing regulation. Exons, intron, splice consensus sequences (GU or AG), U2 branch point (A), polypyrimidine tract (PPT), splicing enhancer/silencer.

Many of the components contributing to accurate intron splicing are already known. These include the splicing regulator, consensus sequence, branch point, exonic/intronic splicing enhancer/silencer sequence, and polypyrimidine tract (Fig. 1)^16^. Therefore, we focused on previously reported *COL4A5* intronic mutations separated from exons. We obtained the sequences of all reported disease-causing *COL4A5* mutations (at intronic positions 10 to 40 bp upstream of the subsequent exon) from The Human Gene Mutation Database (HGMD) and added one from our case^3,17–21^. Using a splicing reporter minigene system, we introduced the different mutations and investigated splicing pattern changes. Finally, we speculate on the mechanisms of the splicing pattern changes using evidence from *in silico* analyses.

## Results

**Figure 2:**
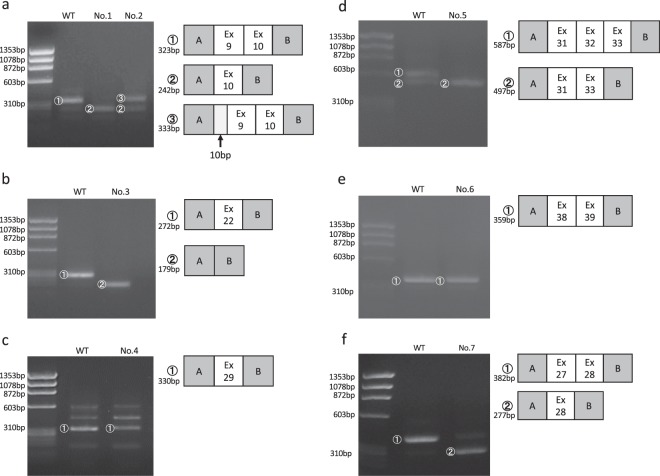
Minigene assay transcript analysis. Electrophoresis results and schematic transcript analysis from the minigene constructs. The direct sequence is shown in Supplementary Fig. 2. (**a**) Wild type (WT) exhibited a single band (full) and No. 1 (case) exhibited a single band (exon 9 skipping), while No. 2 (case) exhibited double bands (exon 9 skipping and 10-bp insertion). (**b**) WT exhibited a single band (full) and No. 3 (case) exhibited a single band (exon 22 skipping). (**c**) Both WT and No. 4 (case) exhibited many bands, which were difficult to compare. (**d**) WT exhibited double bands (full and exon 32 skipping) and No. 5 (case) exhibited a single band (exon 32 skipping). (**e**) Both WT and No. 6 (case) exhibited single bands (full). (**f**) WT exhibited a single band (full) and No. 7 (case) exhibited a single band (exon 27 skipping). No cropped pictures are used in this figure.

The minigene assay revealed aberrant splicing in five of seven variants. We observed exon skipping in four variants (No. 1, No. 3, No. 5 and No. 7), both exon skipping and a 10-bp insertion in one variant (No. 2), and no change in one variant (No. 6) (Table 1, Fig. 2 and Supplementary Fig. 2). Analysis of minigene construct No. 4 (intron 28-14T>A), containing introns 28–29, was not possible. The inserted sequence produced numerous nonspecific bands, making it difficult to assess the splicing pattern. Among three of the seven variants (No. 2, No. 5 and No. 7), our *in vitro* assay results were identical to the previously reported patient transcripts (Table 1). In these cases, mRNA samples were extracted from skin fibroblasts (No. 2), hair roots (No. 5) or peripheral blood leukocytes (No. 7). The results of *in silico* analysis are shown in Table 1. A marked decrease in the original acceptor site score was found for No. 2, No. 4 and No. 5. This suggests that the corresponding changes made it more difficult for the original acceptor site to function in exonic recognition. Novel splicing acceptor sites were created in No. 2, No. 4 and No. 7, suggesting the possibility of insertion or deletion. Indeed, No. 2 leads to a 10-bp insertion in addition to exon 9 skipping (Supplementary Fig. 3). In three variants, reduced polypyrimidine tract scores were observed (No. 3, No. 5 and No. 6). The polypyrimidine tract is preceded by a branch point sequence and is important for downstream exonic recognition. A reduced polypyrimidine tract score may lead to impaired exonic recognition. However, minigene analysis showed no change in splicing pattern in No. 6, even though a reduced polypyrimidine tract score was observed. Of the six cases in which *in vitro* results were obtained, in four cases (No. 1, No. 2, No. 3 and No. 5) the results could be predicted to some extent by the *in silico* results, while in No. 6 and No. 7 prediction from the *in silico* results was difficult (Table 1). At present, this *in silico* analysis is insufficient to predict the precise splicing pattern. In all cases, no branch point alteration was observed.

**Table 1:** mRNA, *in vitro* (minigene) and *in silico* assays.

	gDNA mutation	mRNA	*In vitro* (minigene)			*In silico*			Reference
			Genetic region cloned	Cloning via	Result	HSF (original ASS)	HSF (novel ASS)	SVM-BPF (PPT score)	
No. 1	IVS8-17 T>G	N/A	Intron8–10	Restriction and ligation	Ex9 skipping	7.54 → 6.44		→	Nagel *et al*.^17^
No. 2	IVS8-12 G>A	Ex9 skipping/ 10 bp ins	Intron8–10	Restriction and ligation	Ex9 skipping/ 10 bp ins	7.54 → 2.74	/→3.4	→	Wang *et al*.^18^
No. 3	IVS21-20 T>A	N/A	Intron21–22	Restriction and ligation	Ex22 skipping	8.09 → 5.99		↓	Bekheirnia *et al*.^3^
No. 4	IVS28-14 T>A	N/A	Intron28–29	In-fusion reaction	N/A	3.87→/	/→4.68	→	Weber *et al*.^19^
No. 5	IVS31-10 T>G	Ex 32 skipping	Intron30–33	In-fusion reaction	Ex32 skipping	5.68→/		↓	King *et al*.^20^
No. 6	IVS37-11 C>A	N/A	Intron37–39	In-fusion reaction	No change	6.45 → 4.92		↓	Martin *et al*.^21^
No. 7	IVS26-18 A>G	Ex27 skipping	Intron26–28	Restriction and ligation	Ex27 skipping	12.03 → 10.7	/→6.97	→	Our case

IVS: intron, N/A: not available, Ex: exon.

HSF: Human Splicing Finder, ASS: acceptor site score, SVM-BPF: SVM-BPfinder, PPT: polypyrimidine tract.

## Discussion

This is the first report comprehensively exploring *COL4A5* splicing patterns in variants assumed to be pathogenic in intronic regions 10 to 40 bp upstream of exons. Although XLAS is a monogenic disease caused by *COL4A5*, pathogenic *COL4A5* mutations are not detected in all clinically diagnosed XLAS patients. In such cases, intronic mutations or exonic synonymous mutations can cause aberrant splicing^4–6^. Moreover, 15–60% of pathogenic mutations cause genetic disease through pre-mRNA splicing abnormalities^22^. Therefore, a renewed focus should be placed on intronic variants that can cause splicing abnormalities.

Recently, splicing has been focused on as a target of treatment. Modifying the splicing pattern is an important goal of some molecular therapies^23^. Oligonucleotide-based therapies such as Nusinersen or Eteplirsen have been approved by the FDA^24,25^. Clarification of the involvement of splicing in disease pathogenicity can lead to the development of treatments. For XLAS, we recently reported the phenotype–genotype correlation restricted to cases with *COL4A5* splicing abnormalities. These results suggested the possibility of oligonucleotide therapy leading to exon skipping in patients with XLAS^5^. Taken together, these results show that determining the involvement of splicing in the pathogenicity of inherited kidney diseases is important and may inform treatment approaches.

When nucleotide changes are detected in a gene, it is important to determine whether or not they are pathogenic. This is often difficult, particularly when variants are found in introns. To assess the pathogenicity of intronic variants, it is important to conduct transcript analysis. Tools for *in silico* analysis, such as the Human Splicing Finder (http://www.umd.be/HSF3/), can be used to predict the effect of transcriptional variants and may aid in predicting the disruption of the original consensus splice sites. We previously reported that, among 41 families with *COL4A5* variants inducing aberrant splicing, 19 were completely compatible with the Human Splicing Finder results. Of the remaining families, 17 yielded data that enabled prediction of splicing defects, a precise novel splice site could not be predicted, and the remaining five families yielded insufficient data for the prediction of splicing abnormalities^5^. In this study, No. 6 showed decreasing polypyrimidine tract scores, but *in vitro* results showed no difference between this patient and the control. Therefore, the limitation of *in silico* analysis lies in accurately predicting splicing patterns.

The most accurate method to predict pathogenicity is to conduct transcriptional analysis using the patients’ affected organ tissues. However, obtaining kidney specimens to observe tissue-specific gene expression is always difficult. In addition to renal tissue, peripheral blood lymphocytes, skin biopsies, hair roots and urine-derived cells from patients have also been reported as adjuncts for the diagnosis of AS^5,6,14,18,20,26–29^. It may be possible to determine the pathogenicity of a mutation by examining the mRNA extracted from each sample. However, this approach is suboptimal as transcripts are not stable within tissues, and abnormal transcripts can be quickly degraded by nonsense-mediated mRNA decay^30^. Instead, it has been reported that it is effective to examine the expression of type IV collagen α5 chain in the hair root, which can be collected noninvasively, but the sensitivity and specificity have not been fully established^14^. Therefore, there remains a serious need for another system to assess the pathogenicity of variants of uncertain significance.

Recently, a splicing assay with a hybrid minigene approach has been established as a relatively fast and accurate means to identify splicing aberrations and to study their underlying functional mechanisms^10–15,31^. This approach has been validated by reports of the same results being obtained from *in vivo* and *in vitro* studies^10–12,32^. In our study, in three of the seven patient transcript analyses, the obtained results were completely consistent with our *in vitro* assay results. In one case, our minigene system did not work, possibly because of the low original acceptor site score. In addition, the results of No. 5 minigene showed normal and skipping bands in the WT, but only skipping bands in a patient (Fig. 2d). The possibility of identifying abnormal splicing bands in the WT has already been reported in other *COL4A5* studies with minigene assay^5,15^, which can lead to false positive results of aberrant splicing. Therefore, the *in vitro* results should be evaluated in comparison with controls, bearing in mind variation in splicing patterns among different tissues and differences between *in vitro* and *in vivo* conditions.

*In silico* analysis assisted the interpretation of *in viv*o or *in vitro* analysis results, but was insufficient to predict the splicing pattern by itself. Three intronic variants analyzed by *COL4A5* minigene assay have been reported so far^5,14,15^. Among them, the coexistence of exon skipping and a 43-bp deletion was detected in one case. It was possible to predict exon skipping by *in silico* analysis from reduced branch point scores in that case; however, the 43-bp deletion could not be predicted. Furthermore, although exon skipping occurred in the remaining two cases, their splice site scores decreased only slightly, supporting the difficulty of predicting exon skipping by *in silico* analysis itself (Supplementary Table 1). However, although it is difficult to predict the splicing pattern, clarifying the mechanisms of aberrant splicing by *in silico* analysis allows us to understand how to correct the splicing pattern to weaken or ameliorate the pathogenicity. Combining *in vivo* and/or *in vitro* and *in silico* analyses can thus be a powerful tool for assessing pathogenicity and for the development of appropriate therapeutic approaches.

In conclusion, our splicing assay with hybrid minigene makes it possible to assess whether the mutation in question causes aberrant splicing. In addition, *in silico* tools can predict the aberrant splicing mechanisms. Our system is useful to increase the accuracy of genetic tests through determining the pathogenicity of intronic mutations and may help inform treatment discovery strategies.

## Methods

### *In vitro* splicing assay

To create hybrid minigene constructs, we used the H492 vector that we developed previously, which is based on the pcDNA 3.0 mammalian expression vector and contains a multicloning site (Invitrogen, Carlsbad, CA, USA) (Supplementary Fig. 1)^10^. We cloned DNA fragments containing a couple of exons and introns around the target variant in the *COL4A5* gene using classical restriction and ligation methods or In-fusion cloning methods, as shown in Table 1. As for No. 1–3 and No. 7, cloning was performed by classical restriction and ligation methods using *Nhe*I and *BamH*I. As for No. 4–6, we used infusion cloning methods with the HD Cloning Kit (Takara Bio Inc., Tokyo, Japan), in accordance with the manufacturer’s instructions. As for No. 7, the patients’ gDNA was available, so gDNA of the patients and WT was cloned. As for No. 1–6, because the patients’ gDNA was not available (just the sequence was reported), we initiated cloning with WT gDNA and then introduced mutations by site-directed mutagenesis using PrimeSTAR mutagenesis basal kit (Takara Bio Inc.), in accordance with the manufacturer’s instructions. The primers used are shown in Supplementary Table 1.

The hybrid minigenes were confirmed by sequencing and transfected into HEK293T and HeLa cells using Lipofectamine^®^ 2000 (Thermo Fisher Scientific, Waltham, MA, CA). Total RNA was extracted from cells after 24 h using the Rneasy^®^ Plus Mini Kit (QIAGEN, GmbH, Hilden, Germany). Total RNA (1 µg) was reverse-transcribed using RNA to cDNA EcoDry Premix (Double Primed) (Takara Bio Inc). PCR was performed using a forward primer corresponding to a segment upstream of exon A (YH307: 5′-ATTACTCGCTCAGAAGCTGTGTTGC-3′) and a reverse primer complementary to a segment downstream of exon B (YH308: 5′-CTGCCAGTTGCTAAGTGAGAGACTT-3′). PCR products were analysed by electrophoresis on an 1.5% agarose gel using φX174-Hae III digest marker and direct sequencing.

### *In silico* splicing assay

We predicted splicing domain strength in each variant, using Human Splicing Finder (http://www.umd.be/HSF3/) and SVM-BP Finder (http://regulatorygenomics.upf.edu/Software/SVM_BP/)^33,34^. As for potential splice sites, scores obtained using MaxEnt Scan matrix are shown.

### Compliance with ethical standards

All procedures were reviewed and approved by the Institutional Review Board of Kobe University School of Medicine.

## Supplementary information

Supplementary Dataset 1

## Data Availability

The datasets generated and/or analysed during the current study are available from the corresponding author on reasonable request.
