# Supplementary material for: Determination of the pathogenicity of known COL4A5 intronic variants by in vitro splicing assay
Source: Sci Rep. 2019 Sep 3;9:12696. doi: 10.1038/s41598-019-48990-9 (PMC6722096; doi:10.1038/s41598-019-48990-9)
Supplement: Supplementary file 1 — Supplementary Dataset 1 [file 41598_2019_48990_MOESM1_ESM.docx]

**Supplementary data**

**Title:**

**Determination of the pathogenicity of known *COL4A5* intronic variants by *in vitro* splicing assay**

**Tomoko Horinouchi, Kandai Nozu, Tomohiko Yamamura, Shogo Minamikawa, China Nagano, Nana Sakakibara, Koichi Nakanishi, Yuko Shima, Naoya Morisada, Shinya Ishiko, Yuya Aoto, Hiroaki Nagase, Hiroki Takeda, Rini Rossanti, Hiroshi Kaito, Masafumi Matsuo, Kazumoto Iijima**

**Supplementary Figure 1**.


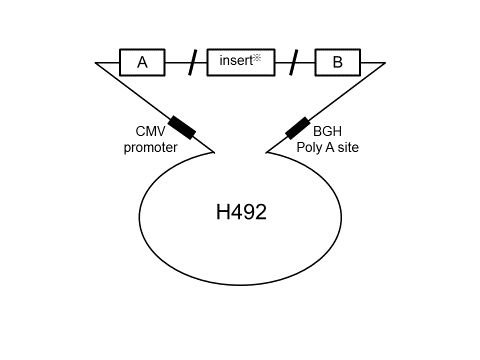


**Supplementary Figure 2.**


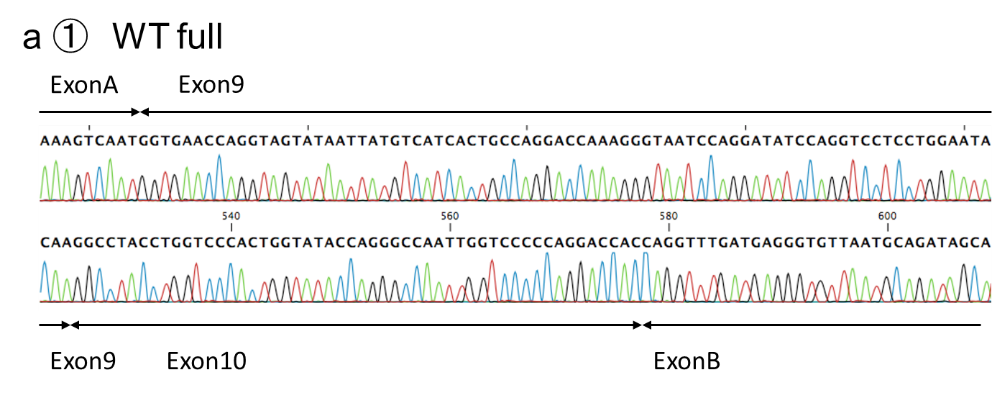


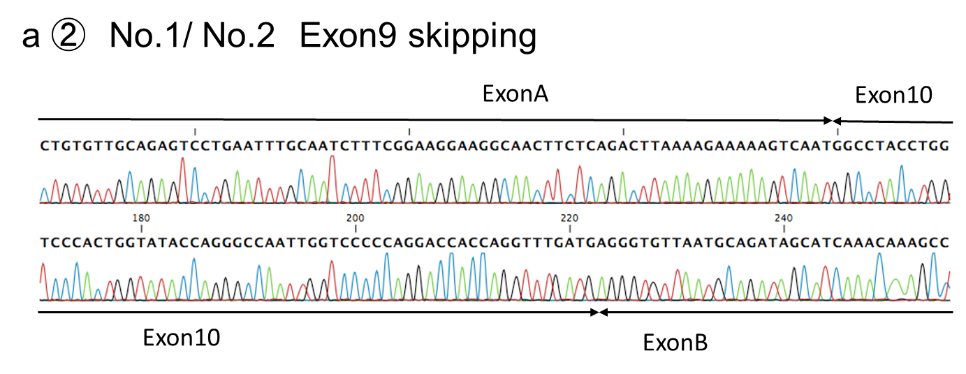


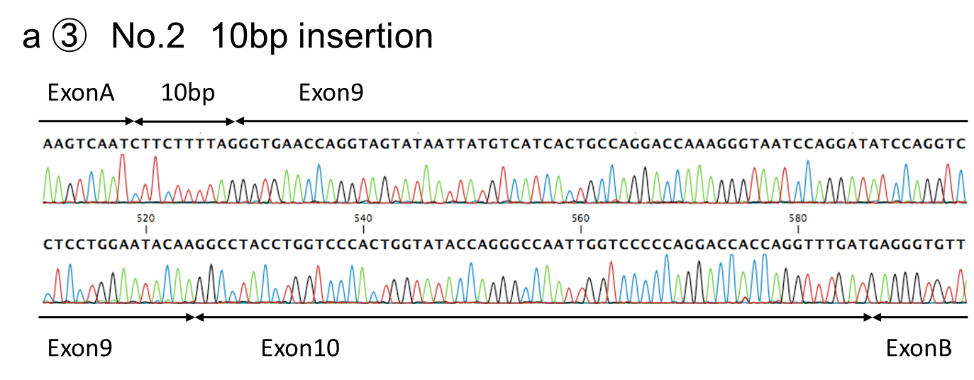


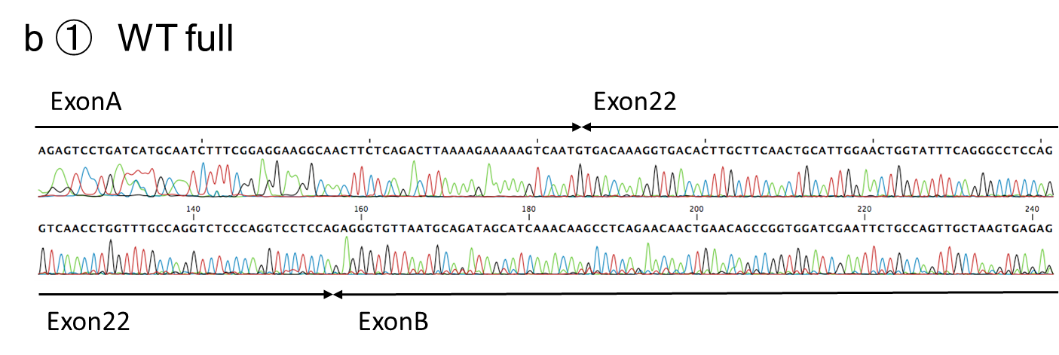


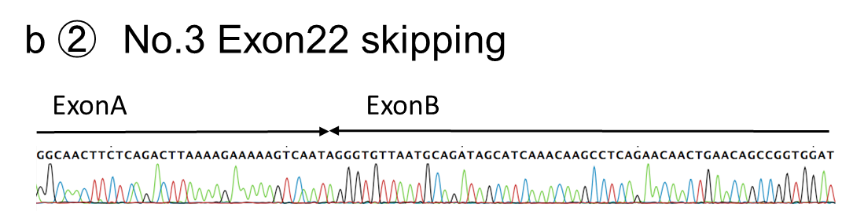


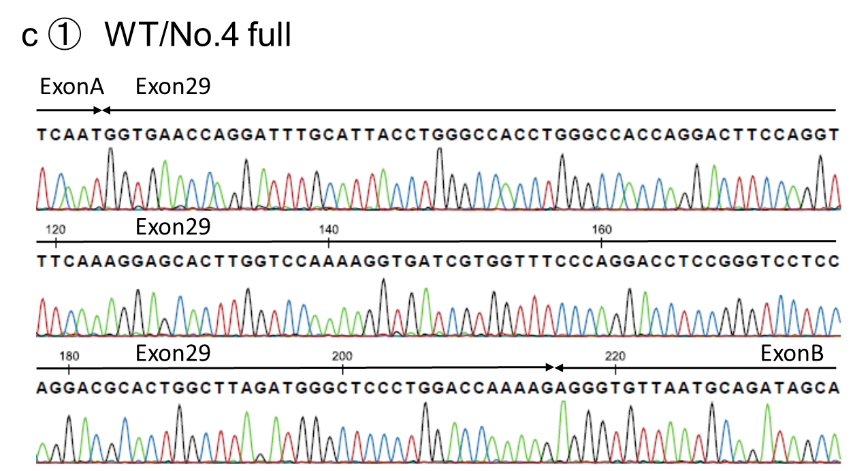


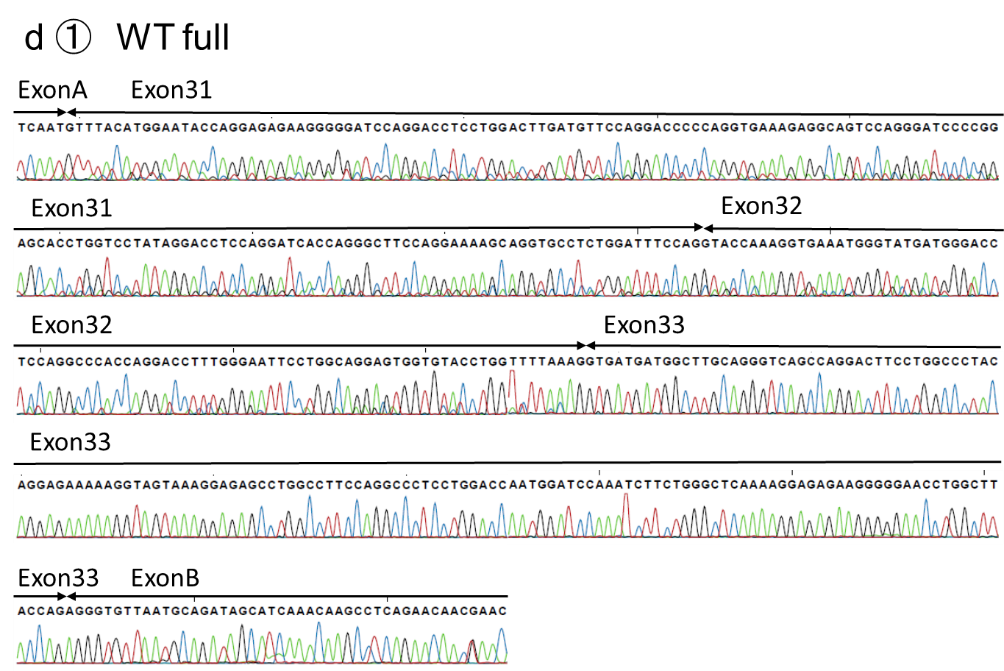


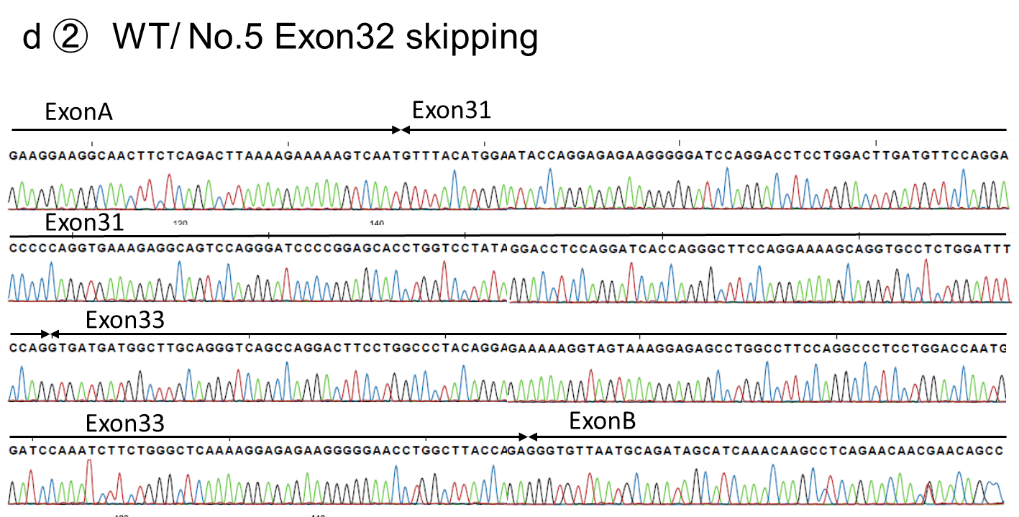


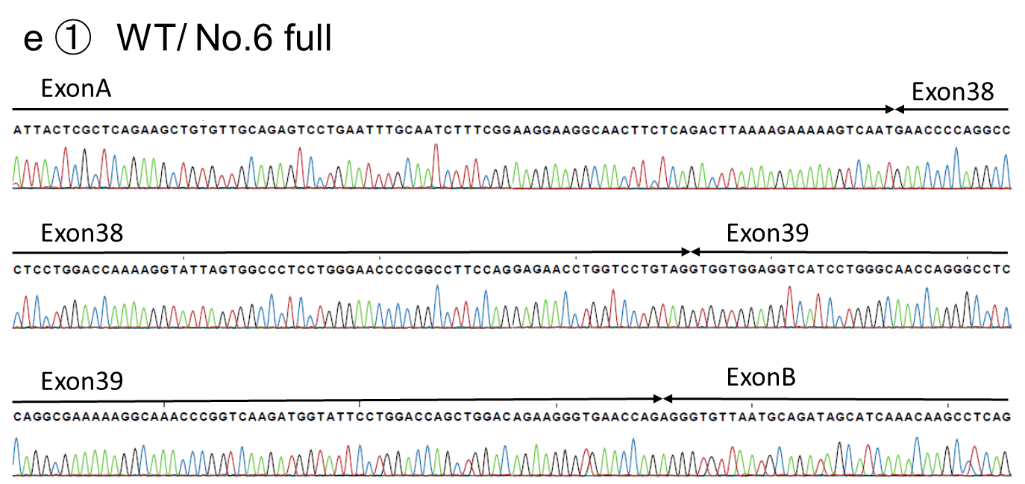


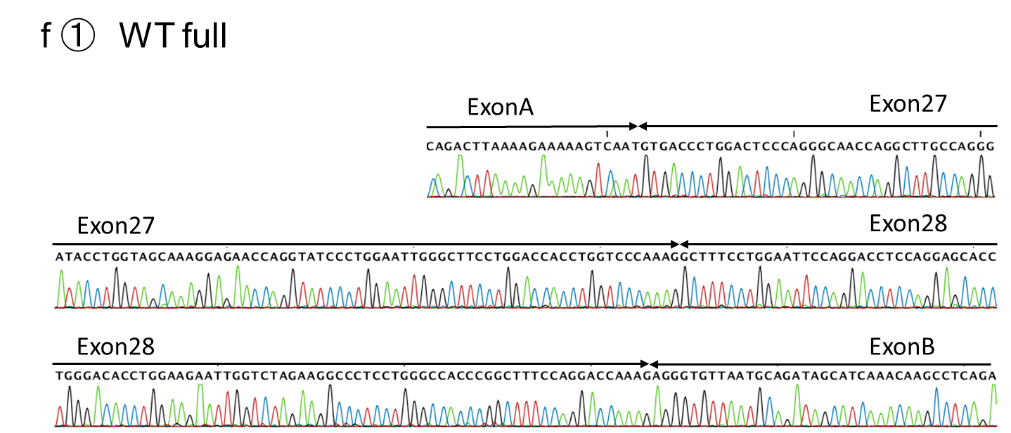


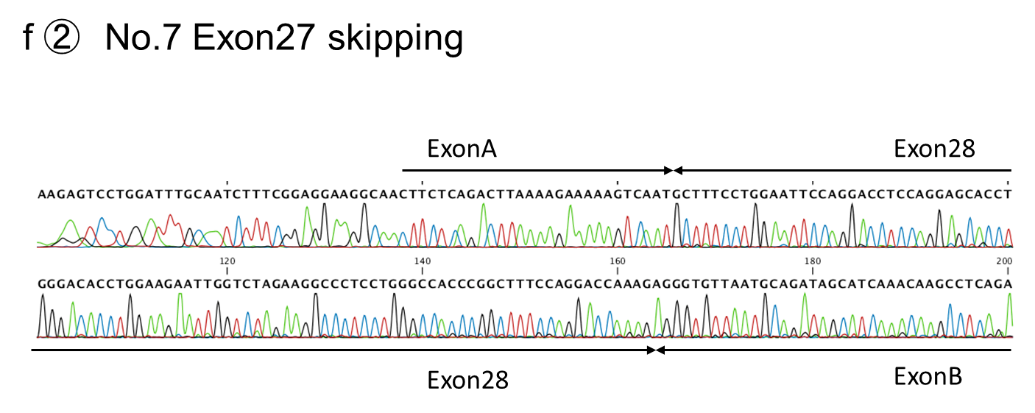


**Supplementary Figure 3.**


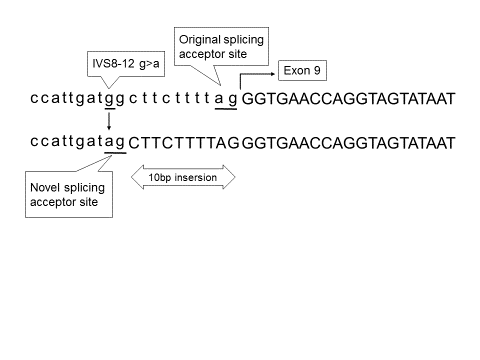


**Figure legends**

**Supplementary Figure 1. Schema for the hybrid minigene**

H492 vector has two cassette exons, A and B, between which is a multicloning site. H492 vector also has cytomegalovirus (CMV) enhancer–promoter and a bovine growth hormone gene (BGH) polyadenylation site.

**Supplementary Figure 2. Direct sequencing for transcript analysis from the minigene constructs**

The numbers correspond to those in Figure 1.

a.① The single band shown in the wild type (WT) corresponded to exon 9 and exon 10 between exon A and exon B; the splicing pattern was normal.

a.② The single band shown in No. 1 and the lower band shown in No. 2 corresponded to only exon 10 between exon A and exon B; exon 9 skipping occurred.

a.③ The upper band shown in No. 2 corresponded to a 10-bp insertion and exon 9 and exon 10 between exon A and exon B; a 10-bp insertion occurred.

b.① The single band shown in WT corresponded to exon 22 between exon A and exon B; the splicing pattern was normal.

b.② The single band shown in No. 3 directly connected exon A and exon B; exon 22 skipping occurred.

d.① The upper band shown in WT corresponded to exon 31, exon 32 and exon 33 between exon A and exon B; the splicing pattern was normal.

d.② The lower band shown in WT and the single band shown in No. 5 corresponded to exon 31 and exon 33 between exon A and exon B; exon 32 skipping occurred.

e.① The single band shown in WT and No. 6 corresponded to exon 38 and exon 39 between exon A and exon B; the splicing pattern was normal.

f.① The single band shown in WT corresponded to exon 27 and exon 28 between exon A and exon B; the splicing pattern was normal.

f.② The single band shown in No. 7 corresponded to exon 28 between exon A and exon B; exon 27 skipping occurred.

**Supplementary Figure 3. Alignment of *COL4A5* exon 9 and its boundaries (No. 2)**

The single-nucleotide substitution activates a novel splicing acceptor site and induces a 10-bp insertion.

**Supplementary Table 1**

*In silico* analysis of *COL4A5* cases for which *in vitro* (minigene) results have been reported

| gDNA mutation | mRNA | *In vitro* (minigene) | *In silico* | | | | Reference |
| --- | --- | --- | --- | --- | --- | --- | --- |
|  |  |  | HSF (original ASS) | HSF  (novel ASS) | SVM-BPF (PPT score) | HSF  (Branch point) |  |
| IVS28-40 A>G | N/A | Ex29 skipping/ 43bp del | N/A | N/A | ↓ | 59.3→29.6 | Chiereghin et al. 2017 |
| IVS24-6 T>G | N/A | Frequent skipping of Ex25 | 12.9→11.2 | / | → | No change | Malone et al.  2017 |
| c.548dupG (Ex10) | Exon10 skipping | Ex10 skipping | 6.8→6.3 | / | → | No change | Horinouchi et al. 2018 |

IVS: intron, N/A: not available, Ex: exon

HSF: Human Splicing Finder, ASS: acceptor site score, SVM-BPF: SVM-BPfinder, PPT: polypyrimidine tract

**Supplementary Table 2**

The primers for cloning

No. 1 F: GCAGCTAGCGGCCACTTCCTCATTTTCC

No. 1 R: CGTGGATCCTCTGAAATGGCCAGAATTGA

No. 2 F: GCAGCTAGCGGCCACTTCCTCATTTTCC

No. 2 R: CGTGGATCCTCTGAAATGGCCAGAATTGA

No. 3 F: GCAGCTAGCCTGGAACAGTTCTTGGCACA

No. 3 R: CGTGGATCCGCTTCAGGTATTAGAAACAAGAGCA

No. 4 Fragment F: CCGTGCTTTGTTAGCTTTCCCTACAAGGGTAGTC

No. 4 Fragment R: TCGATGTTAACGCTAACCTTCACCTCCCAAGTTC

No. 4 Vector F: TAGCGTTAACATCGATATCCGG

No. 4 Vector R: GCTAACAAAGCACGGAGTTTAC

No. 5 Fragment F: AGCGTTAACATCGATTGCATCTTTGGCTTTTTATTC

No. 5 Fragment R: AACCAGGATCCGGATAGTGGTTAGGTAAAGCTCG

No. 5 Vector F: ATCCGGATCCTGGTTTGA

No. 5 Vector R: ATCGATGTTAACGCTAGC

No. 6 Fragment F: AGCGTTAACATCGATAGTCCAGCAAGAGGAATGG

No. 6 Fragment R: AACCAGGATCCGGATAAACATATATTCTATTTCCGATG

No. 6 Vector F: ATCCGGATCCTGGTTTGA

No. 6 Vector R: ATCGATGTTAACGCTAGC

No. 7 F: GCAGCTAGCGTACAGTGGCGTGATGTTGG

No. 7 R: CGTGGATCCTTGCTCCAAATGTGACCTTG

The primers for mutagenesis

No. 1 F: ACTTCCAGTGATGGCTTCTTTTAGGG

No. 1 R: GCCATCACTGGAAGTTCTAAGACACA

No. 2 F: CATTGATAGCTTCTTTTAGGGTGAAC

No. 2 R: AAGAAGCTATCAATGGAAGTTCTAAG

No. 3 F: ATTTGTTAGTTATTATGATTTCACTA

No. 3 R: TAATAACTAACAAATTTTACTGAAAG

No. 4 F: TTTGTCAAGTGTATGCTCAAGGGTGA

No. 4 R: CATACACTTGACAAAACACAACAAAA

No. 5 F: ATTGATAGTCTTCAAAGGTACCAAAG

No. 5 R: TTGAAGACTATCAATAAGTAATGTTTG

No. 6 F: TTGAGCTATTTACTCTAGGAACCCC

No. 6 R: GAGTAAATAGCTCAATTTAAAAATG
